# Women are underrepresented in fields where success is believed to require brilliance

**DOI:** 10.3389/fpsyg.2015.00235

**Published:** 2015-03-11

**Authors:** Meredith Meyer, Andrei Cimpian, Sarah-Jane Leslie

**Affiliations:** ^1^Department of Psychology, Otterbein University, WestervilleOH, USA; ^2^Department of Psychology, University of Illinois at Urbana-Champaign, ChampaignIL, USA; ^3^Department of Philosophy, Princeton University, PrincetonNJ, USA

**Keywords:** gender, stem, lay theories of success, field-specific ability beliefs, diversity in academia

## Abstract

Women’s underrepresentation in science, technology, engineering, and mathematics (STEM) fields is a prominent concern in our society and many others. Closer inspection of this phenomenon reveals a more nuanced picture, however, with women achieving parity with men at the Ph.D. level in certain STEM fields, while also being underrepresented in some *non*-STEM fields. It is important to consider and provide an account of this field-by-field variability. The field-specific ability beliefs (FAB) hypothesis aims to provide such an account, proposing that women are likely to be underrepresented in fields thought to require raw intellectual talent—a sort of talent that women are stereotyped to possess less of than men. In two studies, we provide evidence for the FAB hypothesis, demonstrating that the academic fields believed by laypeople to require brilliance are also the fields with lower female representation. We also found that the FABs of participants with college-level exposure to a field were more predictive of its female representation than those of participants without college exposure, presumably because the former beliefs mirror more closely those of the field’s practitioners (the direct “gatekeepers”). Moreover, the FABs of participants with college exposure to a field predicted the magnitude of the field’s gender gap above and beyond their beliefs about the level of mathematical and verbal skills required. Finally, we found that beliefs about the importance of brilliance to success in a field may predict its female representation in part by fostering the impression that the field demands solitary work and competition with others. These results suggest new solutions for enhancing diversity within STEM and across the academic spectrum.

## Introduction

**FIGURE 1:**
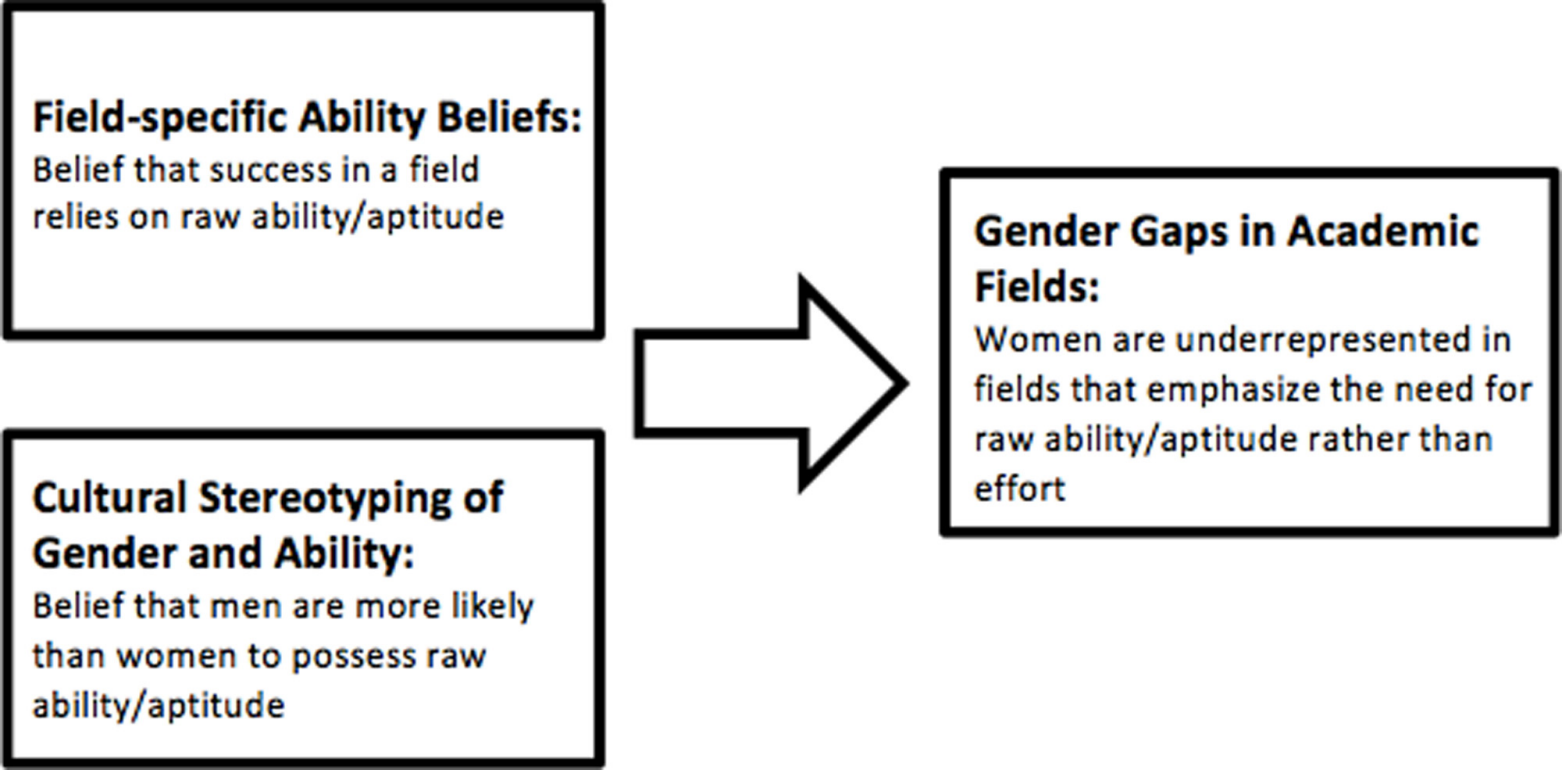
**Diagram of the field-specific ability beliefs (FAB) hypothesis**.

A recent article in *Scientific American Mind* begins: “Try this simple thought experiment. Name 10 female geniuses from any period of history. Odds are you ran out of names pretty quickly” (Upson and Friedman, 2012, p. 63). The thought experiment can be adapted: try to name 10 female figures in popular culture who—like Sherlock Holmes, Dr. House, or Will Hunting—are characterized by their innate brilliance, their raw intellectual firepower. As before, one rapidly runs out of names. Whatever the cause, the message is clear: women are not culturally associated with such inherent gifts of genius (Bennett, 1996, 1997, 2000; Tiedemann, 2000; Rammstedt and Rammsayer, 2002; Furnham et al., 2006; Kirkcaldy et al., 2007; Upson and Friedman, 2012; Lecklider, 2013; Stephens-Davidowitz, 2014). The consequences of this stereotype are likely wide-ranging. In the current study, we focus on one of these consequences, asking whether such a pervasive cultural message might have a role in shaping individuals’ academic and career paths. Specifically, if it is widely believed that men tend to possess more intellectual ability than women, then women may be discouraged from entering into fields that are thought to require this ability. We call this the field-specific ability beliefs (FAB) hypothesis (**Figure 1**): *the more a field is believed to require raw brilliance, the fewer the women* (Leslie and Cimpian et al., 2015). We test this hypothesis in the context of gender gaps in academia, investigating whether these gaps are predicted by how much laypeople assume that success in various fields rests on raw ability.

Gender disparity in academia has been a generative topic of research for many years, with contemporary focus on this issue largely centering on men’s and women’s participation in (natural) sciences, technology, engineering, and mathematics (STEM). The general phenomenon is clear: on average, female representation in STEM fields (particularly those that are math-intensive) is lower than in the social sciences and humanities (SocSci/Hum). Though the magnitude of this gap has largely decreased across the last several decades, the difference is still reliable, prompting a number of efforts to explain it (for reviews, see Ceci and Williams, 2007; Ceci et al., 2009, 2014; Hill et al., 2010).

The low number of women in STEM is indeed of real concern. However, it is also important to observe that there is at least as much variation in female representation *within* STEM and SocSci/Hum as there is *between* them. For instance, when examining the number of recent doctoral degrees earned by women in the U.S. in 30 different fields (**Table 1**), STEM fields are characterized by female representation ranging from just under 20% (physics) to over 50% (molecular biology; National Science Foundation [NSF], 2011). An even larger range is observed within SocSci/Hum fields, with women earning fewer than 35% of doctoral degrees in philosophy and economics, yet over 75% in art history. Indeed, the range of variation is so wide that many STEM fields feature *higher* female representation at the Ph.D. level than many SocSci/Hum fields. Given this large variation within STEM and SocSci/Hum considered separately, it is apparent that expanding the focus of inquiry to include gender gaps in both STEM and SocSci/Hum might provide new insights into the problem of female underrepresentation. In the current study, we adopt such a broad focus, examining whether the FAB hypothesis can account for the field-by-field variability observed across the entire academic spectrum.

**Table 1:** Percent of American Ph.D.’s earned by women in 2011* in science, technology, engineering, and mathematics (STEM) and Social science/Humanities fields.

STEM Field	% of Ph.D.’s who are Female	Social science/Humanities field	% of Ph.D.’s who are Female
Physics	18.0	Music theory and composition	15.8
Computer science	18.6	Philosophy	31.4
Engineering	22.2	Economics	34.4
Mathematics	28.6	Middle Eastern studies	38.1
Astronomy	29.2	Classics	41.8
Earth sciences	36.2	Political science	43.1
Chemistry	37.8	History	45.0
Statistics	41.6	Archeology	52.3
Biochemistry	45.4	Linguistics	59.2
Neuroscience	49.4	Anthropology	59.6
Evolutionary biology	49.8	Spanish/Spanish literature	59.9
Molecular biology	54.4	Comparative literature	60.9
		Sociology	61.3
		English literature	62.4
		Communication studies	64.2
		Education	69.3
		Psychology	72.1
		Art history	76.8

*Data from 2011 NSF Survey of Earned Doctorates.

### Initial Evidence for the FAB Hypothesis

In a recent study, we sought to test whether the FABs held by *academics* could predict the wide field-by-field variability observed in female representation across both STEM and non-STEM fields (Leslie and Cimpian et al., 2015). We gathered responses from a sample of over 1800 professors, graduate students, and post-doctoral researchers from research-intensive universities across the U.S. in 30 different fields (12 STEM, 18 SocSci/Hum; **Table 1**). We first asked participants to report on their beliefs regarding what was required for success in their own field, focusing on assessing beliefs about the relative importance of intrinsic, stable ability vs. effort and practice (see Dweck, 2000, 2006). We then used these items to provide a metric of field-level ability beliefs; each field received a FAB score expressing average endorsement of ability vs. effort across individuals within a given field (with higher scores indicating more emphasis on raw ability). Results included three important findings. First, FABs were strongly negatively associated with female representation (as measured by proportion of U.S. Ph.D. degrees earned by women; National Science Foundation [NSF], 2011), providing initial broad support for the hypothesis: there were fewer women in fields believed to require stable, raw talent. Second, ability beliefs were predictive of female representation over and above whether a field was STEM or SocSci/Hum, suggesting that the FAB hypothesis can account well for the wide variability observed even within the two categories of fields. Finally, the FAB hypothesis outperformed a number of other constructs often theorized to contribute to gender gaps in academia, including field-specific variation in work-life balance (e.g., Ferriman et al., 2009), selectivity (e.g., Hedges and Nowell, 1995), and reliance on skills related to systemizing vs. empathizing (e.g., Baron-Cohen, 2002).

### The Present Research

#### Do Laypeople’s Beliefs Predict Female Representation?

Results from Leslie and Cimpian et al. (2015) suggest that women are underrepresented in fields whose practitioners consistently endorse the idea that success rests on brilliance. The current study extends existing work on the FAB hypothesis by exploring what *non-academics* believe is required for success in a variety of fields. We hypothesize that the FABs endorsed by academics will be shared, at least to some extent, by people outside of academia as well. This is an important extension of the FAB framework because, to the extent that these beliefs are shared by the general public, they could influence women’s career choices in a much broader variety of contexts than the beliefs of academics *per se*. If these beliefs pervade our society, then—in combination with the stereotypes against women’s intellectual abilities—they could lead a variety of individuals (parents, teachers, peers, etc.) to see women as somewhat unsuited for “brilliance-required” domains. Even in the absence of such biased treatment, widely shared ability beliefs similar to those previously identified in academics could lead young women to doubt that they could succeed in brilliance-focused disciplines and thus to decide against pursuing careers in them. Our main prediction is thus that laypeople’s beliefs, like those of academics, will predict female representation: the more a field is believed to require intellectual brilliance, the fewer the women.

We can also formulate a more detailed hypothesis here: people with more exposure to the fields in question (e.g., via college classes) will have FABs that predict female representation more precisely than the beliefs of people with less exposure. We expect this to be the case because the FABs of those with more exposure to a discipline will likely be more similar to those of the practitioners of that discipline, and are thus more likely to be similar to the kinds of beliefs that students will encounter and absorb as they start to consider higher education and careers in these fields (Leslie and Cimpian et al., 2015). In Study 1, we tested the prediction that college-exposed individuals’ ability beliefs would better predict gender gaps in representation by first dividing our participants into those who had college exposure to a field and those who did not, and then exploring whether the beliefs of the college-exposed group predict female representation at a more fine-grained level.

A final aim of Study 1 was to address an alternative explanation for the hypothesized relationship between ability beliefs and female representation. As we have argued, underlying the main predictions described above is our claim that FABs influence women’s academic and career choices. However, might laypeople’s beliefs be simply inferred from their pre-existing knowledge about the proportion of women in the different fields? For instance, our participants—particularly those who have had college experience in the relevant fields and thus have had the opportunity to witness gender disparities firsthand—might rely on stereotypes against women’s intellectual abilities to arrive at the conclusion that fields with few women must require high levels of such abilities, whereas fields with many women must not. To address this possibility, we asked participants to estimate the proportion of women in the fields under investigation, and assessed whether participants’ ability beliefs still predicted female representation independent from these estimates. If so, this would undermine the possibility that participants simply infer their ability beliefs from their estimates of the field’s diversity.

#### Assessing Beliefs Beyond FAB

There are surely many other dimensions that vary among fields and influence the gender breakdown of the people who participate in them, and we do not claim that FAB is the only factor in determining academic gender gaps. Indeed, as we observed at the outset, other such factors have been evaluated extensively in prior studies (for reviews, see Ceci and Williams, 2007; Ceci et al., 2009, 2014; Hill et al., 2010). Although an exhaustive evaluation of these additional factors is outside the scope of the current studies, we take up this issue within the framework of evaluating other *beliefs* about what is required for success in the fields under investigation. In particular, Study 2 examines two questions. First, do field-specific beliefs about the importance of intellectual brilliance reduce to beliefs about specific types of skills required for success? Specifically, do they reduce to beliefs about the degree to which mathematical and verbal skills are required for individual fields? Second, is the relationship between FABs and female representation mediated by beliefs about what kinds of work (solitary vs. collaborative; competitive vs. cooperative) are required?

Our first question addresses a potential alternative explanation for the predictive power of FABs. A critic might note that the extent to which mathematics is involved in a field appears to be particularly predictive of whether women are underrepresented or not: fields that are math-intensive attract and retain fewer women, with math-intensive STEM fields (e.g., engineering, math, or physics) characterized by the most extreme gender disparities (in comparison to STEM fields that are less math-intensive, like the life sciences, which often feature parity or even a predominance of women; National Science Foundation [NSF], 2011). The smaller number of women in math-intensive fields may be due in part to the cultural belief that math is “for” males, a belief that appears to emerge as early as elementary school and may contribute to women’s reduced interest in careers that require it (Fredericks and Eccles, 2002; Herbert and Stipek, 2005; Cvencek et al., 2011). In light of this evidence, one might ask: is it possible that the “intellectual brilliance” at the heart of the FAB hypothesis is just another way of referring to mathematical aptitude, which is also popularly conceived as a fixed, innate quantity? That is, might it be the case that people’s FABs simply reduce to their beliefs about how much individual fields require math over other kinds of skills (e.g., verbal skills)?

Results from Study 1 could bear on this question. If, as hypothesized we find that FABs are capable of predicting female representation across a variety of fields, including those unlikely to be thought of as drawing on mathematical skills (such as most social sciences and humanities disciplines), it is unlikely that these beliefs are *merely* capturing people’s beliefs about field-specific mathematical requirements. However, it is important to more directly establish whether FABs are distinct from beliefs about the importance of mathematics. To do so, in Study 2 we tested whether beliefs about raw ability and brilliance predict unique variance in gender gaps, beyond that predicted by people’s beliefs about how much individual fields rely on mathematical and verbal ability.

Finally, it is worth noting that, as in Study 1, college exposure may matter. Participants with college experience likely have more nuanced, differentiated beliefs both about which fields require mathematical skills and about which fields require intrinsic ability. We thus hypothesized that when looking specifically at individuals with college exposure, FABs would independently predict female representation over and above beliefs about math and verbal skills, supporting the idea that FAB can account for female representation across the academic spectrum.

Next, we turn to the issue of potential beliefs that may mediate the relationship between FABs and female representation. In particular, we explore the possibility that people’s beliefs about the importance of brilliance vs. effort for success in a field give rise to differentiated perceptions of the kind of atmosphere that field promotes. We focused our exploration on two important aspects of a field’s atmosphere that (1) could be plausibly inferred based on the field’s presumed emphasis on brilliance, and that (2) men and women have diverging attitudes toward: namely, the extent to which the field requires *competition* (vs. collaboration) and *solitary work* (vs. group work; e.g., Lippa, 1998; Niederle and Vesterlund, 2007; Diekman et al., 2010; Gupta et al., 2013; Lippa et al., 2014). There are several reasons why “brilliance-required” fields might also be presumed to require competition and solitary work. If a field values intellectual prowess, it is reasonable to expect that it would also encourage *displays* of that sort of ability, which might in turn encourage competition between individual practitioners. After all, it is only by comparing one’s ability against others (by participating in contests, engaging in aggressive debates, being harshly critical of others’ perceived mistakes, etc.) that one can reveal how brightly one’s intellectual ability shines. Working with others in cooperative contexts, on the other hand, would make it hard to assess whose talent was responsible for any ultimate success attained, so this type of collaborative work may be assumed to be rare within fields that prize brilliance. The inference that brilliance-requiring fields involve solitary, and often competitive, work is also likely to be supported by pervasive cultural tropes that portray brilliance and genius as qualities that a person possesses and displays in isolation rather than as part of a team of collaborators (e.g., Shenk, 2014). In turn, these inferences about the nature of the work environment in a field may influence whether young men and women consider careers in it because males and females are socialized to place different value on communal vs. agentic goals and on collaborative vs. competitive interactions. In other words, the downstream inferences licensed by FABs may be part of the reason why these beliefs are predictive of gender gaps^1^. We tested this hypothesis in Study 2.

### Summary of Predictions

Study 1 examined two main predictions, one broad and one more specific. Broadly speaking, we expected that there would be a relationship between laypeople’s FABs and female representation, such that fields believed to require brilliance would have fewer women. At a greater level of specificity, we expected that college exposure would differentiate the predictive power of FABs, such that the beliefs of those exposed to the fields during college would be particularly predictive. Finally, Study 1 also examined whether ability beliefs independently predicted female representation above and beyond people’s *estimates* of female representation (suggesting that any observed relationship between ability beliefs and actual female representation did not emerge simply because individuals constructed FABs from their beliefs about female representation).

Study 2 was designed to replicate the main findings of Study 1, and to extend the inquiry into additional beliefs that might relate to gender disparities. We made two predictions. First, we predicted that FABs would *not* reduce to people’s beliefs about mathematical skill, particularly when examining beliefs from individuals with college exposure in the field. Second, fields that are believed to require raw ability should also be perceived as requiring solo work and competition; in turn, these perceptions should predict gender gaps, with fewer women obtaining Ph.D.’s in fields assumed to demand high levels of solo work and competition. In other words, we expected that beliefs about solo work and competition would mediate (at least partially) the observed association between FAB and gender breakdowns.

## Study 1

### Method

#### Participants

Participants included 307 individuals recruited via Amazon’s Mechanical Turk (MTurk), an online crowd-sourcing platform^2^^,^^3^ . Only participants reporting themselves as living in the U.S. and with prior MTurk approval rates of 90% or above were included. Participants were compensated $0.75 for survey completion. Data were excluded from an additional 48 individuals who (1) failed to complete the survey, (2) answered an attention-check question incorrectly, (3) had IP addresses indicating they were outside the U.S., and/or (4) had IP addresses indicating they had completed similar studies in the past.

#### Materials and Procedure

To avoid participant fatigue, we created three versions of the survey, each of which contained 10 of the 30 fields under investigation. (Fields were identical to those examined in our original study of academics (Leslie and Cimpian et al., 2015)). Fields were chosen to represent a broad spectrum of social sciences, humanities, and STEM disciplines. Approximately equal numbers of subjects participated in the three versions, and assignment was random (Version 1, *n* = 103; Version 2, *n* = 101; Version 3, *n* = 103). Each version included three humanities subjects, three social science subjects, and four STEM subjects. Each survey contained four questions assessing FABs about each of the 10 fields (from Leslie and Cimpian et al., 2015; **Table 2**). Questions were presented individually in random order with all 10 fields listed beneath each question. Participants indicated their agreement with the statement as it applied to each field using a 7-point Likert scale (1 = strongly disagree to 7 = strongly agree, with eight as an option to indicate “don’t know”). Two attention-check questions were also included to ensure that participants were attending to the task.

**Table 2:** Survey items for Study 1 and Study 2.

**Field-specific ability beliefs**	
	Being a top scholar of [field] requires a special aptitude that just can’t be taught.
	If you want to succeed in [field], hard work alone just won’t cut it; you need to have an innate gift or talent.
	With the right amount of effort and dedication, anyone can become a top scholar in [field]. (R)
	When it comes to [field], the most important factors for success are motivation and sustained effort; raw ability is secondary. (R)
	To succeed in [field] you have to be a special kind of person; not just anyone can be successful in it. (in Study 2 only.)
	People who are successful in [field] are very different from ordinary people. (in Study 2 only.)
**Estimate of female representation (Study 1)**	
	Please provide your best guess or estimate to this question: in the recent past, what percentage of doctoral (Ph.D.) degrees from American universities do you think have been earned by women in [field]?
**Verbal and mathematical ability (Study 2)**	
	Top-level success in [field] depends to a large extent on one’s verbal ability.
	Top-level success in [field] depends to a large extent on one’s mathematical ability.
**Solo and competitive work (Study 2)**	
	[Field] is a field in which you spend a lot of time working by yourself rather than being around other people.
	[Field] is a field in which competition with others is much more common than collaboration.

(R) indicates items that were reverse scored.

Responses to all items except estimate of female representation were given on a 7-point scale (1 = strongly disagree to 7 = strongly agree), with an additional option for “don’t know.” Responses for estimate of female representation were given on a 10-point scale, with each point representing a 10% increment.

Next, a series of questions asked about participants’ academic exposure to the 10 fields, including whether they had had (1) a high school class, (2) a college class, and/or (3) a graduate-level class in each of them. Participants were also asked to estimate how many women had received American doctoral degrees in each field in the recent past, with 10 response options corresponding to 10% intervals ranging from 0 to 100%. A final set of questions asked about demographic information (gender, age, ethnicity, and race).

For each field, we calculated FAB scores by averaging scores across participants from the four ability belief questions. Higher scores indicated more emphasis on brilliance. Three separate FAB scores were calculated: (1) All Participants’ FAB (using data from all participants except those with graduate level experience in the field)^4^, (2) College Exposure FAB (using data from participants who had taken college, but not graduate level, courses in the field) and (3) No College Exposure FAB (using data from participants who had taken neither college nor graduate courses in the field). The four items had high internal reliability (for all participants, α = 0.90; for College Exposure, α = 0.93; for No College Exposure, α = 0.89).

### Results and Discussion

Study 1 tested two main predictions. First, we expected that participants’ FABs would be correlated with female representation regardless of participants’ level of direct prior exposure with the fields (via courses). Second, we predicted that beliefs held by individuals with college experience would nevertheless be predictive of female representation at a *finer-grained level* than those of people with no college experience. In particular, we expected that the College Exposure, but not the No College Exposure, FAB scores would predict female representation even after taking into account a gross STEM vs. non-STEM distinction between fields, which would speak to the ability of the College Exposure FAB scores to predict the complex field-by-field variability in female representation observed within these broad domains. Finally, we examined whether beliefs of college-exposed and non-college-exposed individuals predicted actual female representation independent of participants’ *estimates* of female representation. If so, this would rule out the possibility that ability beliefs predicted female representation for the trivial reason that they were inferred from participants’ pre-existing knowledge about gender disparities.

**FIGURE 2:**
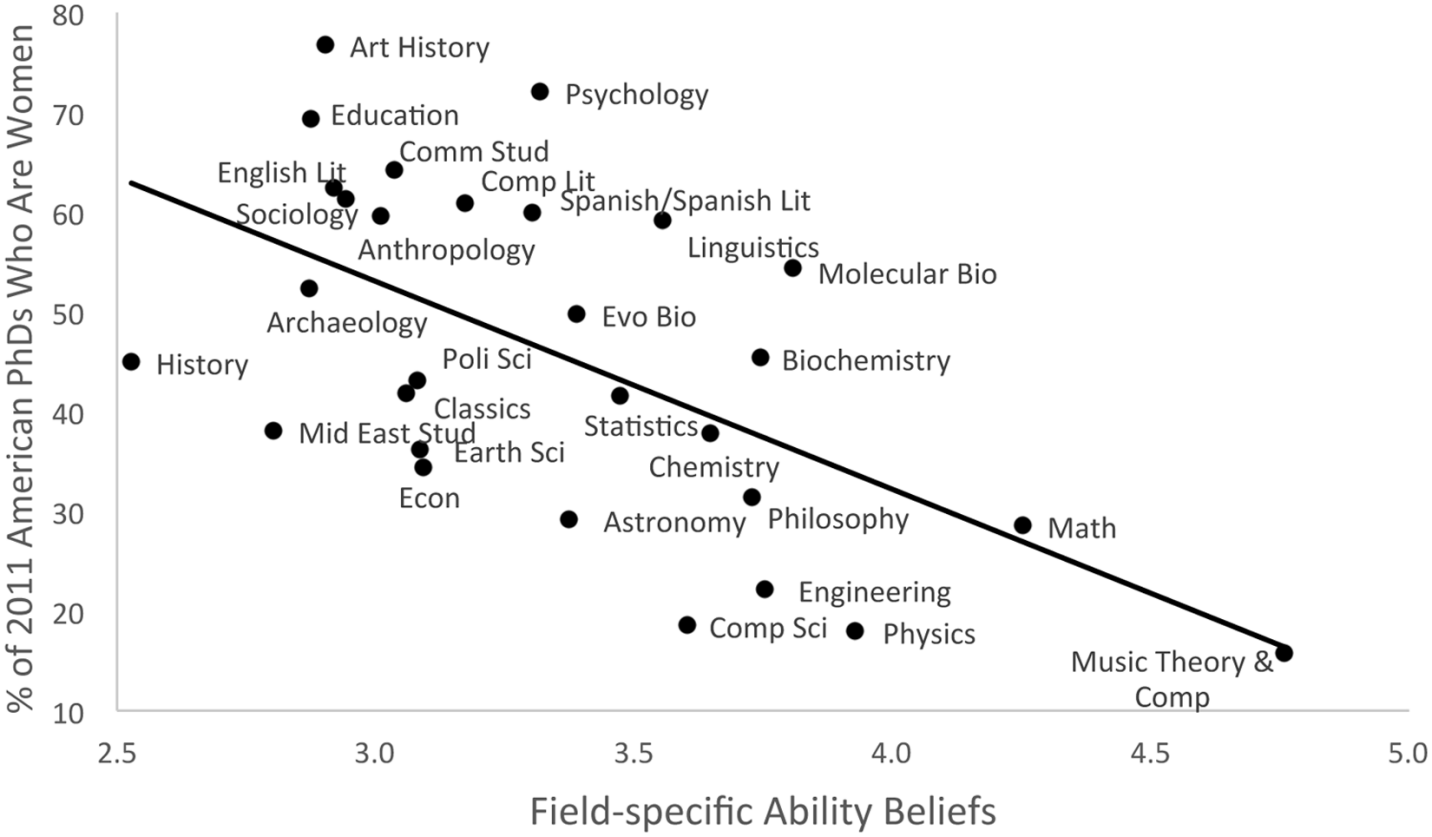
**The relationship between FABs (all participants) and female representation (Study 1)**.

To assess our first prediction, we examined the correlation between FABs and female representation. Any fields for which we received fewer than 10 participants in either the no-college-experience or college-experience samples were removed from the analysis; estimates based on so few participants would likely be unreliable. This resulted in 29 fields being retained for analysis. (The single removed field was neuroscience; only seven individuals reported college experience with this field.) As predicted, fields believed to require brilliance had lower female representation, *r*(27) = -0.59, *p* = 0.001 (**Figure 2**).

To address the second prediction, we separately examined beliefs held by people with college exposure and those held by people without college exposure. Beliefs of both groups were significantly negatively associated with female representation: College Exposure scores, *r*(27) = -0.67, *p* < 0.001, and No College Exposure scores, *r*(27) = -0.51, *p* = 0.005. Steiger’s *z* score comparison (Lee and Preacher, 2013) indicated that College Exposure scores were more strongly associated with female representation than No College Exposure scores, *z* = 2.09, *p* = 0.037, providing initial support for the prediction that college-exposed individuals’ beliefs would relate more strongly with representation. We then investigated whether the ability beliefs of these two groups predicted female representation above and beyond whether a field was STEM vs. a social science/humanities discipline (i.e., non-STEM). Two separate multiple regression analyses were performed with female representation as the dependent variable and two predictors: a STEM/non-STEM indicator variable and either (1) College Exposure FAB scores or (2) No College Exposure FAB scores. These analyses indicated that, as hypothesized, the FABs held by participants with college exposure to the fields were uniquely predictive of female representation, above and beyond whether the fields were in STEM or SocSci/Hum (β = -0.44, bootstrapped *p* = 0.013), whereas the beliefs of participants without college exposure were not (β = -0.15, bootstrapped *p* = 0.449).

Finally, we added college-exposed and non-college-exposed participants’ estimates of female representation as predictors to the two regressions above. Consistent with our argument, the FABs of college-exposed participants remained a significant predictor of actual female representation even when adjusting for these participants’ estimates of female representation (β = -0.41, bootstrapped *p* = 0.043; see **Table 3**). In contrast, the beliefs of participants without college exposure were not a significant predictor of female representation in this model (β = -0.30, bootstrapped *p* = 0.257; see **Table 3**). Thus, it is not the case that college-exposed participants’ ability beliefs are predictive of gender gaps across academia simply because they are derived from prior knowledge of such gaps.

**Table 3:** Regressions predicting female representation using field-specific ability beliefs and estimates of female representation of participants with college experience (CE; Upper) and with no college experience (NCE; Lower), Study 1.

Predictor			*R*^2^	*F*	*p*
	β	*p*	0.686	15.87	< 0.001
STEM indicator	0.07	0.686			
Estimate of female representation (CE)	0.58	0.001			
Field-specific ability beliefs (CE)	-0.41	0.043			

	β	*p*	0.55	10.37	<0.001
STEM indicator	-0.22	0.340			
Estimate of female representation (NCE)	0.44	0.023			
Field-specific ability beliefs (NCE)	-0.30	0.257			

*n* = 29, df (4,24).

We considered one final alternative interpretation, which applies particularly to the findings obtained with college-exposed individuals. Perhaps College Exposure FAB scores emphasize brilliance for fields where there are few women just because (1) men may be more likely than women to believe that brilliance is required for success, and (2) more men in the current sample may have taken college classes in disciplines where women are typically underrepresented. In other words, disciplines with lower female representation may have higher College Exposure FAB scores for the simple reason that male participants’ brilliance-focused ability beliefs are overrepresented in our sample for these disciplines. Consistent with this possibility, college-exposed men’s scores (*M* = 3.56, *SD* = 0.55) were indeed higher than college-exposed women’s scores (*M* = 3.18, *SD* = 0.64), *t*(28) = 4.02, *p* < 0.001, suggesting that men placed more emphasis on raw ability. In addition, our sample contained proportionately more college-exposed men in fields with lower female representation at the Ph.D. level, *r*(27) = -0.66, *p* < 0.001. To test whether these differences could explain our main result, we calculated a gender-balanced FAB score for each field by computing the average scores for men and women separately within fields, and then averaging these two gender-specific scores. This measure adjusts for the differential representation of college-exposed males and females across fields, giving the two groups an equal say in determining the FAB score for each field. If the current alternative explanation were correct, this gender-balanced score should no longer be predictive of female representation. However, when we entered the gender-balanced FAB score in place of the original FAB score in the regression including both STEM status and estimated female representation, it still predicted female representation (β = -0.35, bootstrapped *p* = 0.069). Thus, the main results described above were not merely a byproduct of men’s brilliance-oriented beliefs inflating the College Exposure FAB scores of fields with fewer women.

In sum, the results of Study 1 lend clear support to the predictions we derived from the FAB model: women are less likely to be represented in fields believed to require stable, innate ability. Furthermore, as predicted, the field-specific beliefs of people with college experience in our fields were predictive of female representation at a more detailed level than were the beliefs of those without college experience. To speculate, perhaps initially people hold a global belief that disciplines in the STEM family require innate skill; as a result, the predictive power of these initial, inchoate ability beliefs is mostly captured by the STEM vs. non-STEM distinction. It is only after exposure to the particularities of the fields and the beliefs of their practitioners that FABs take on independent predictive power in relation to female representation.

Study 2 provides an opportunity to replicate the above findings, and to further explore how gender breakdowns are related to field-differentiated beliefs about the types of skills and work that are required. Two predictions are central to Study 2. First, we expect that the FABs of participants with college experience will predict unique variance in female representation, above and beyond their beliefs about the role of mathematical or verbal skills. Second, we predict that participants’ assumptions about how much solitary and competitive work is required by individual fields will mediate the relationship between FABs and female representation.

## Study 2

### Method

#### Participants

Participants included 302 individuals recruited via Amazon’s MTurk, using the same inclusion criteria as in Study 1. Participants were compensated $0.95 for survey completion. Data were excluded from an additional 53 individuals who met one or more of the exclusion criteria used in Study 1: (1) failing to complete the survey, (2) answering an attention check question incorrectly, (3) having an IP address suggesting residence outside the U.S., and/or (4) having IP addresses indicating completion of similar studies (including our Study 1) in the past.

#### Materials and Procedure

As in Study 1, three versions of the survey were created, each of which contained the same subsets of 10 of the 30 fields under investigation. Approximately equal numbers of subjects participated in the three versions, and assignment was random (Version 1, *n* = 101; Version 2, *n* = 103; Version 3, *n* = 98). Surveys included the same four FAB items as in Study 1, along with two additional, broader questions on this topic (**Table 2**). These questions were added to further assess FABs, with the goal of using more accessible language while still providing a sensitive index of participants’ beliefs about innate ability vs. effort. Two items were also included to address people’s beliefs about the extent to which verbal and mathematical skills are required, and two final items were included to assess beliefs about whether competition and solitary work are important for success in a field (**Table 2**).

As in Study 1, items were presented individually in random order with all 10 fields listed beneath each item. Participants again indicated their agreement with the statement as it applied to each of the 10 fields using a 7-point Likert scale (1 = strongly disagree to 7 = strongly agree, with eight as an option to indicate “don’t know”). Two attention-check questions were also included. The survey then ended with questions assessing high school, college, and graduate level exposure to each of the 10 fields, along with several demographic questions. (These questions were all identical to those in Study 1).

We calculated FAB scores by averaging scores across the six items, and then averaging within fields to create field-level scores. Three separate FAB scores were calculated reflecting (1) All Participants’ FAB (using data from all participants except those with graduate level experience in the field), (2) College Exposure FAB (using data from participants who had taken college, but not graduate level, courses in the field), and (3) No College Exposure FAB (using data from participants who had taken neither college nor graduate courses in the field). Scores for the six ability beliefs questions had high internal reliability (for all participants, α = 0.89; for College Exposure, α = 0.93; for No College Exposure, α = 0.87). Deletion of the last two items added for Study 2 did not improve scale reliability, indicating it was appropriate to include them as part of the FAB scale.

### Results and Discussion

To explore whether Study 2 replicated the key finding that FABs predict female representation, we again examined correlations between FAB and percentage of female Ph.D. recipients. As before, fields with fewer than 10 participants reporting either college or no college exposure were removed. This resulted in 27 fields being retained for analysis. (Middle Eastern studies, neuroscience, and archeology were removed because they had College Exposure *n*s of 8, 4, and 3, respectively.) Replicating findings from Study 1, FAB scores were negatively associated with female representation when examining belief scores of all participants, *r*(25) = -0.63, *p* < 0.001, as well as when examining College Exposure scores, *r*(25) = -0.65, *p* < 0.001, and No College Exposure scores, *r*(25) = -0.54, *p* = 0.004. Although College Exposure scores were more strongly associated with female representation than No College Exposure scores, this difference was not significant according to Steiger’s *z* score comparison (Lee and Preacher, 2013), *z* = 1.13, *p* = 0.258. However, again replicating Study 1, we found that the FABs of participants with college experience predicted female representation even when a STEM indicator variable was added to the regression model as a competitor (β = -0.37; bootstrapped *p* = 0.048); in contrast, the beliefs of those without college experience were not uniquely predictive when the STEM indicator was added (β = -0.15; bootstrapped *p* = 0.48). Thus, beliefs held by college-exposed individuals again predicted female representation better than those of non-college-exposed individuals.

We next examined the relationship between female representation and the extent to which a field is perceived as demanding verbal and mathematical skills. Beliefs about the need for verbal skills were positively associated with female representation: beliefs of all participants, *r*(25) = 0.63, *p* < 0.001; of participants with college exposure, *r*(25) = 0.63, *p* = 0.001; of participants with no college exposure, *r*(25) = 0.65, *p* < 0.001. Beliefs about the need for mathematical skills were negatively associated with female representation: beliefs of all participants, *r*(25) = -0.64, *p* < 0.001; of participants with college exposure, *r*(25) = -0.60, *p* = 0.001; of participants with no college exposure *r*(25) = -0.64, *p* < 0.001.

We then tested our prediction that FABs of individuals with college exposure would predict female representation independently from beliefs about the role of mathematical and verbal skills. If so, this would strengthen the claim that FABs tap into something distinct from people’s beliefs about which fields require mathematical aptitude. To assess this prediction, we added perceptions of the need for verbal and mathematical skill as variables in the two regressions predicting female representation. For the regression testing beliefs of those *with* college experience (**Table 4**), FABs were uniquely predictive of women’s representation, above and beyond STEM status and beliefs about the importance of mathematical and verbal skill, β = -0.39, bootstrapped *p* = 0.085, although this coefficient was only significant at the α = 0.10 level. In contrast, beliefs about the importance of verbal and mathematical ability did not independently predict female representation in this model, *p*s > 0.489. For the regression testing the beliefs of those *without* college experience, no factor was significantly predictive of female representation, *p*s > 0.454 (**Table 4**).

**Table 4:** Regressions predicting female representation using field-specific ability beliefs and beliefs about the importance of verbal and mathematical skill of participants with college experience (CE; Upper) and with no college experience (NCE; Lower), Study 2.

Predictor			*R*^2^	*F*	*p*
	β	*p*	0.52	6.05	0.002
STEM indicator	-0.11	0.747			
Field-specific ability beliefs (CE)	-0.39	0.085			
Verbal skill beliefs (CE)	0.26	0.489			
Mathematical skill beliefs (CE)	-0.06	0.820			

	β	*p*	0.49	5.29	0.004
STEM indicator	-0.21	0.524			
Field-specific ability Beliefs (NCE)	-0.14	0.516			
Verbal skill beliefs (NCE)	0.17	0.614			
Mathematical skill beliefs (NCE)	-0.26	0.454			

*n* = 27, df (4,22).

As in Study 1, we also calculated a gender-balanced FAB score to examine the possibility that differences in male and female participants’ ability beliefs and college experience were driving the effects observed for college-exposed participants. (To reiterate, the possibility being tested here is that College Exposure FAB scores in fields with fewer women are inflated simply because men may have ability beliefs that are more brilliance-oriented and may also be overrepresented in the college-exposure sample for these fields.) Again, the proportion of college-exposed male participants within each field was negatively related to female representation at the Ph.D. level, *r*(25) = -0.41, *p* = 0.03, indicating that college-exposed male participants were more numerous in fields with lower female representation. In this sample, however, college-exposed men’s FAB scores (*M* = 3.70, *SD* = 0.71) were actually lower than college-exposed women’s scores (*M* = 3.85, *SD* = 0.60), though not significantly so, *t*(26) = 1.69, *p* = 0.103. Thus, this alternative explanation is unlikely: college-exposed male participants, though more numerous in fields with fewer women at the Ph.D. level, did *not* differ from college-exposed female participants in their ability beliefs. Nevertheless, we entered a gender-balanced FAB score in place of the original FAB score in the regression model that also included a STEM indicator, beliefs about mathematical ability, and beliefs about verbal ability. As before, ability beliefs were the sole predictor of female representation (β = -0.44, bootstrapped *p* = 0.059). These results strengthen the main claim that ability beliefs are predictive of female representation, above and beyond beliefs about mathematical and verbal skills.

**FIGURE 3:**
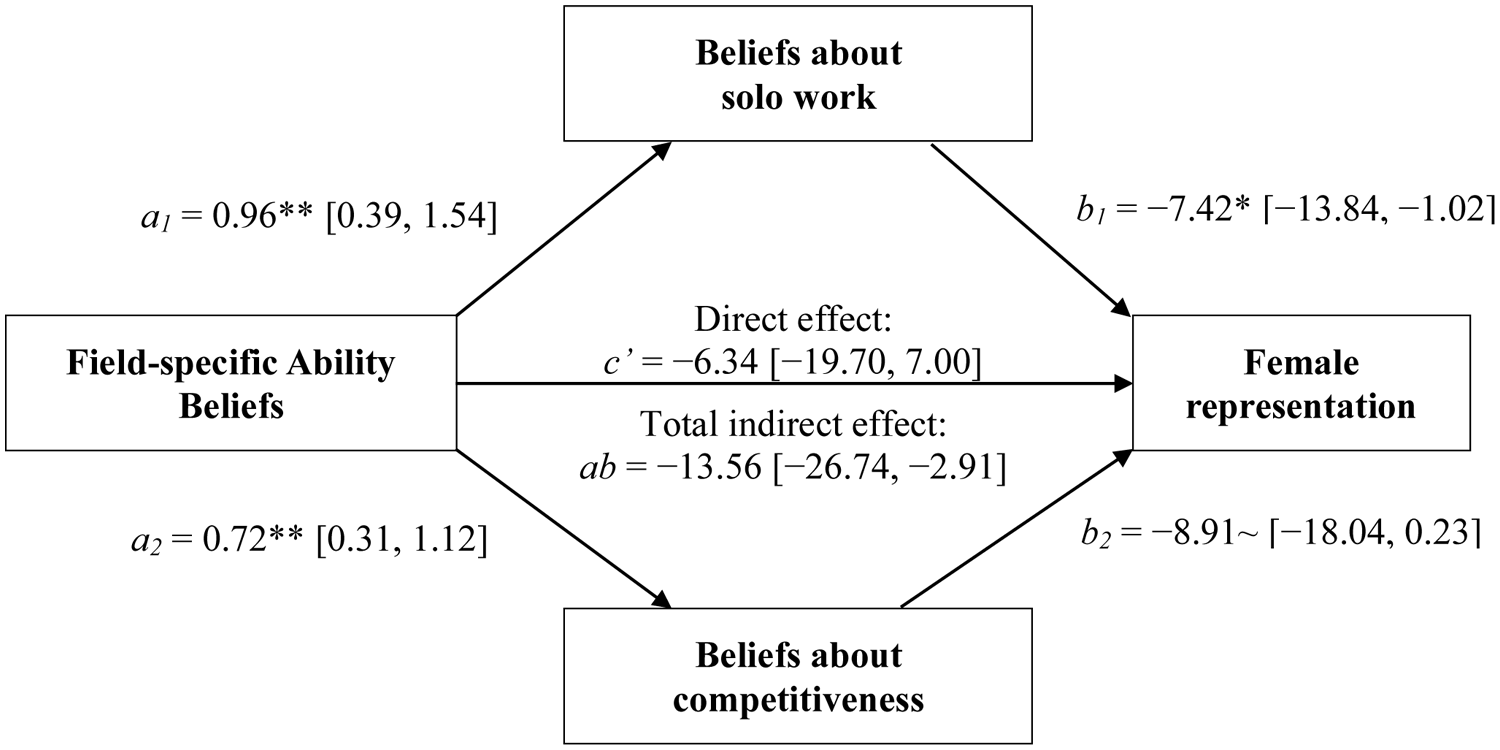
**The indirect pathways linking college-exposed participants’ FABs with women’s representation via participants’ beliefs about the amount of solo work (top) and the level of competitiveness (bottom) required by a field (Study 2).** ∼*p* < 0.10; *p < 0.05; **p < 0.01.

**FIGURE 4:**
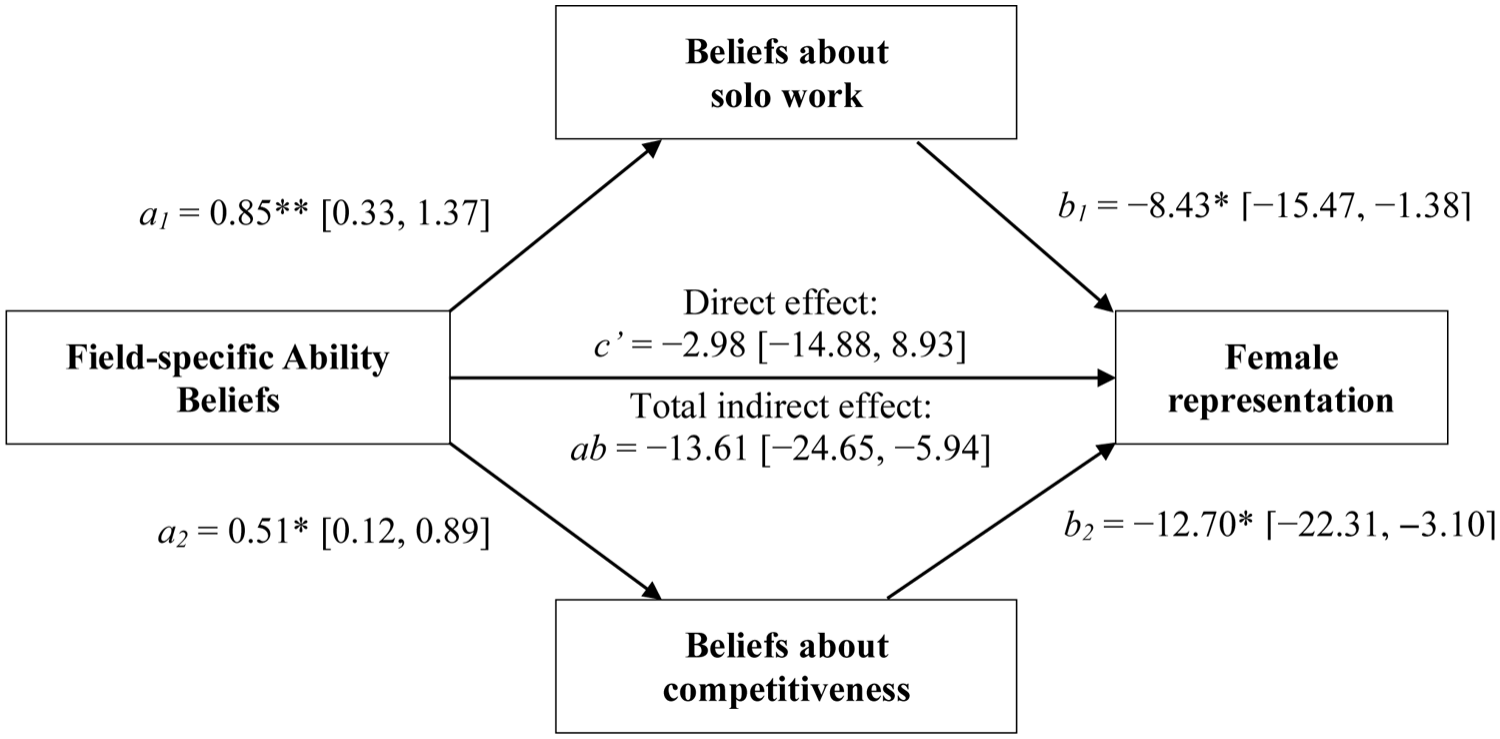
**The indirect pathways linking non-college-exposed participants’ FABs with women’s representation via participants’ beliefs about the amount of solo work **(top)** and the level of competitiveness **(bottom)** required by a field (Study 2).** *p < 0.05; **p < 0.01.

Finally, we tested the prediction that beliefs about solo work and competitiveness would mediate the relationship between FABs and female representation. Consistent with our argument, a bootstrapped (1,000 replications) product-of-coefficients mediation analysis performed with the PROCESS procedure in SPSS 22 (Hayes, 2013) revealed that the relationship between college-exposed participants’ ability beliefs about a discipline and the proportion of female Ph.D.’s in that discipline was significantly mediated by these participants’ ideas about the amount of solo work and the level of competitiveness required by the discipline, ab = -13.56 (-26.74, -2.91). Similar results were obtained when examining beliefs of non-college-exposed participants, ab = -13.61 (-24.65, -5.94)^5^. (For full results of the mediation models, see **Figures 3** and **4**.) Results are thus consistent with the idea that FABs may influence women’s participation in a field in part by influencing their beliefs about what it is like to be a member of that field—in particular, whether one works by oneself or with others, and whether success rests more on competition with colleagues rather than cooperation. Interestingly, this result was observed even within the group who had not had college exposure to the field, which may be because inferences about the nature of the work demanded by various fields are easily drawn from one’s ability beliefs about these fields, no matter how much first-hand experience one has with them.

## General Discussion

Women are underrepresented in many STEM fields, but the pattern of gender distribution is complex, and a substantial amount of variation also exists in non-STEM fields. An important aim of the current studies was to provide an account for the wide variability in female representation across the entire academic spectrum. We maintain that the FAB hypothesis provides such an account. This hypothesis predicts that women will be underrepresented in fields believed to emphasize brilliance and inherent ability as the key to success; this is because women are often stereotyped as lacking the same sort of innate intelligence as men, and thus women will be discouraged from participating in fields to the extent that these fields are perceived as requiring this type of intelligence. Prior research has provided support for the FAB hypothesis within higher academia (Leslie and Cimpian et al., 2015). The current studies extended the focus to an examination of beliefs held by individuals outside academia. The results of our two studies are consistent with the FABs hypothesis: fewer women are involved in fields that laypeople believe to require raw intellectual ability.

Several additional findings from the present studies are worth highlighting. The ability beliefs of individuals who had college-level exposure to the fields in question predicted female representation even when controlling for whether a field was in STEM or not, indicating that college may provide a unique context for refinement and elaboration of beliefs about what fields require for success. Results also suggested that the ability beliefs of participants with college experience are not simply a byproduct of participants’ inferring these beliefs based on their prior knowledge of female representation (Study 1). Further, college-exposed participants’ ability beliefs capture something beyond perceptions of specific types of skills required for success, as FABs of college-exposed individuals did not reduce to beliefs about which fields require mathematical and/or verbal skills (Study 2).

Notably, these findings have important consequences for potential interventions to improve diversity, both in terms of timing and in terms of content. College may be a pivotal experience during which people’s FABs become entrenched, and start to conform to those of their instructors. This highlights the crucial role that college educators play in communicating these maladaptive beliefs—but also suggests that they may be able to play an active role in changing the relevant messages. In particular, our data suggest that instructors who want to promote diversity might aim to minimize discussion of innate talent, regardless of the domain of skills with which it is associated, and instead highlight the importance of effort, practice, and persistence to success in a field. Prior work on individuals’ achievement beliefs suggests that such growth-oriented messages can be relayed in a range of ways: by choice of adjectives (in particular by avoiding words like “brilliant,” “genius,” etc.; , Mueller and Dweck, 1998; Cimpian et al., 2007; Heyman, 2008), by focusing on what the person* has achieved* rather than on the person’s* inherent traits* (Kamins and Dweck, 1999), and by explicitly stating that dedication and effort are paramount (Mueller and Dweck, 1998; Kamins and Dweck, 1999; Blackwell et al., 2007). We expect that practices such as these would be easily implementable by college educators across many fields. It should be borne in mind that the messages that college educators send may not only affect the participation of the women in their classes, but also have more far-reaching impact. As their students—both men and women—may go on to become parents, caregivers, school teachers, etc., they may subtly communicate their own ability beliefs to future generations (e.g., through their own choice of adjectives; Cimpian et al., 2007). This in turn may influence even very young girls’ engagement and educational choices (Cimpian et al., 2014; Leslie et al., 2015).

The current studies also suggest that beliefs about solo and competitive work may mediate the relationship between ability beliefs and female representation. It is possible that this result reflects a process by which ability beliefs influence perceptions of what it is like to work in certain fields, which in turn may influence the participation of women in these fields. Of course, we acknowledge this is not the only possible pathway here; our mediation analyses were designed to test an a priori hypothesis regarding how ability beliefs relate to representation, but they cannot determine directionality. Similarly, causality regarding ability beliefs and female representation cannot be claimed from the current studies due to the correlational nature of the data. However, our theoretical model posits that ability beliefs do drive women’s career and educational choices, and recent experimental manipulations in our lab have provided evidence consistent with this causal claim (e.g., Cimpian et al., 2014). For instance, simply describing a novel educational or professional opportunity as requiring raw talent (vs. dedication) was sufficient to lower women’s—and even young girls’—motivation to pursue it. Thus, there is some independent evidence suggesting that the relationship between ability beliefs and female representation is due to the causal influence of the ability beliefs.

Further investigating the precise pathways by which non-academics’ ability beliefs influence participation is one important topic for future research. To begin, it is worth noting that young men and women often decide whether or not to pursue a field long before interacting with professors, graduate students, or any other active practitioners of that field (e.g., Watt and Eccles, 2008). Indeed, many fields with disproportionately high representation of men at the Ph.D. level see gender disparities in interest as early as elementary school (Lubinski and Benbow, 2006; Ceci and Williams, 2010; Cvencek et al., 2011). From our viewpoint, some of these early differences may be due to the ability beliefs of people outside of academia (teachers, parents, peers, etc.). For example, adults’ FABs, in combination with the stereotype that females are less likely than males to be brilliant, could lead to small differences in the extent to which adults encourage girls’ and boys’ interest in fields believed to require this intellectual trait, the extent to which they provide boys and girls with opportunities to develop their skills in these fields, the extent to which they dwell on boys’ and girls’ achievements in these fields, and so on. Adults are also likely to convey their FABs to the children themselves. Once absorbed, these beliefs might make it more difficult for girls to consider careers in fields believed to require brilliance (again, since the ambient stereotypes portray them as being unsuited for these fields). As well, children might communicate these FABs to their peers, either via explicit statements or more subtly—say, by reacting with surprise to behaviors that are inconsistent with these beliefs. As a result of these multiple parallel processes, young women may be less likely to be interested in “brilliance-required” fields, and those who do pursue them may be less likely to persist and achieve at the same levels as men.

In summary, we have provided support for the FAB hypothesis, demonstrating that women tend to be underrepresented in fields believed to require innate intellectual talent for success. Our data also open up possibilities for future research on the pathways by which ability beliefs influence women’s participation. Finally, these studies point to possibilities for effective interventions. If the practitioners of fields with gender gaps made a concerted effort to highlight the role of sustained, long-term effort in achievement, the gender gaps in these fields may correspondingly be diminished.

## Conflict of Interest Statement

The authors declare that the research was conducted in the absence of any commercial or financial relationships that could be construed as a potential conflict of interest.

## Footnotes

1 We acknowledge that beliefs about solo/competitive work mediating FAB’s relationship with women’s representation represents only one possible causal pathway; it is also possible that people could perceive the solo/competitive nature of a field and then conclude that it requires raw ability. More generally, we also note that there are likely many more factors involved in the pathways that ultimately result in the observed field-by-field variation in women’s representation. More comprehensive exploration of these factors, as well as experimental work, will be needed to definitively establish how FABs influence the observed gender gaps.

2 All human subjects research reported in this paper was approved by the Institutional Review Board of the first author’s home institution.

3 Mechanical Turk offers a convenience sample rather than a fully nationally representative sample. Analyses of American MTurk workers have demonstrated that women are overrepresented, that workers are typically younger and more educated than average, and that Blacks and Hispanics are underrepresented (Berinsky et al., 2012; Paolacci and Chandler, 2014). Thus, we do not claim to be capturing beliefs that are fully representative of the U.S. public. Nevertheless, the diversity of an MTurk sample is arguably higher than that of most samples used in human subjects research (i.e., college samples), and it provides a good source of data for an examination of beliefs held by individuals outside of academia.

4 We excluded data regarding individual fields if they were provided by people reporting graduate-level experience in that field. We did so because we wanted to exclude beliefs held by people with extensive familiarity with the field gained through graduate-level exposure, allowing the focus of the current study to be restricted only to individuals with no college experience vs. college experience with the field.

5 The indirect paths were again significant even when adjusting for beliefs about mathematical skills, both for college-exposed participants, ab = -9.21 (-22.11, -1.73) and for non-college-exposed participants, ab = -9.54 (-19.77, -2.89).
